# Adequate access to healthcare and added life expectancy among older adults in China

**DOI:** 10.1186/s12877-020-01524-9

**Published:** 2020-04-09

**Authors:** Lisha Hao, Xin Xu, Matthew E. Dupre, Aimei Guo, Xufan Zhang, Li Qiu, Yuan Zhao, Danan Gu

**Affiliations:** 1 School of Geographic Sciences, Nanjing Normal University, Nanjing, China; 2 Department of Population Health Sciences, Department of Sociology, & Duke Clinical Research Institute, Duke University, Durham, NC USA; 3 Ginling College, Nanjing Normal University, Nanjing, China; 4 New York, NY USA; 5 Ginling College & School of Geographic Sciences, Nanjing Normal University, Nanjing, China; 6 Independent Researcher, New York, USA

**Keywords:** China, Access to healthcare, Life expectancy, Older adults, Oldest-old, Medical care, Healthcare, Gender differences, Urban-rural differences

## Abstract

**Background:**

Adequate access to healthcare is associated with lower risks of mortality at older ages. However, it is largely unknown how many more years of life can be attributed to having adequate access to healthcare compared with having inadequate access to healthcare.

**Method:**

A nationwide longitudinal survey of 27,794 older adults aged 65+ in mainland China from 2002 to 2014 was used for analysis. Multivariate hazard models and life table techniques were used to estimate differences in life expectancy associated with self-reported access to healthcare (adequate vs. inadequate). The findings were assessed after adjusting for a wide range of demographic factors, socioeconomic status, family/social support, health practices, and health conditions.

**Results:**

At age 65, adequate access to healthcare increased life expectancy by approximately 2.0–2.5 years in men and women and across urban-rural areas compared with those who reported inadequate access to healthcare. At age 85, the corresponding increase in life expectancy was 1.0–1.2 years. After adjustment for multiple confounding factors, the increase in life expectancy was reduced to approximately 1.1–1.5 years at age 65 and 0.6–0.8 years at age 85. In women, the net increase in life expectancy attributable to adequate access to healthcare was 6 and 8% at ages 65 and 85, respectively. In men, the net increases in life expectancy were generally greater (10 and 14%) and consistent after covariate adjustments. In contrast, the increase in life expectancy was slightly lower in rural areas (2.0 years at age 65 and 1.0 years at age 85) than in urban areas (2.1 years at age 65 and 1.1 years age 85) when no confounding factors were taken into account. However, the increase in life expectancy was greater in rural areas (1.0 years at age 65 and 0.6 years at age 85) than in urban areas (0.4 years at age 65 and 0.2 years at age 85) after accounting for socioeconomic and other factors.

**Conclusions:**

Adequate access to healthcare was associated with longer life expectancy among older adults in China. These findings have important implications for efforts to improve access to healthcare among older populations in China.

## Background

Access to healthcare is a fundamental social determinant of health that has been targeted by the World Health Organization to improve the availability and timeliness of quality healthcare [1]. Numerous studies have shown that timely and/or adequate access to healthcare promotes better health and well-being [2, 3] and that inadequate access to care has been associated with greater psychological distress [4], lower levels of physical health [5], higher rates of rehospitalization [6], and overall higher risks of morbidity and mortality [3, 7]. Moreover, a large body of literature has demonstrated the correlates and consequences of access to healthcare in a variety of settings and populations [7, 8]. At older ages, adequate access to healthcare is especially critical to maintaining health and preventing the onset (or exacerbation) of diseases through routine preventive care and treatments [5, 9]. Older adults who lack access to healthcare are consequently less likely to receive early diagnoses, timely treatments, and other resources for healthcare that ultimately put them at higher risks of death [10].

With the rapid growth of aging populations around the world [11], it would be valuable to investigate to what extent the timely and/or adequate access to healthcare among older adults promotes health and longevity, especially among older adults in developing countries where healthcare resources are usually limited [12, 13]. Although China has made remarking progress in its economic growth, the per capita income is still relatively low due to its large population base [14, 15]. China today has the largest population of older adults in the world, and thus would be a good case to examine the contribution to health or life expectancy for adequate access to healthcare.

China has been implementing a dual system of social welfare in urban and rural areas to provide nearly universal healthcare to its population since the 1950s [2]. Although the dual-system of healthcare is slowly eroding, the system still plays an important role and consists of several components [16, 17]. In urban areas, the system implemented a free public medical care scheme since the 1950s that provided free healthcare services to its employees in the public sector and/or their family members [5]. In the 1990s, the urban medical scheme was reformed and subsequently split into two systems around 2010—the Urban Employer-sponsored Medical Scheme (UEMS) and the Urban Resident Medical Scheme (URMS, for urban residents without UEMS) [18–20]—although some oldest-old adults were still covered by free public medical care. The UEMS covers urban employees or retirees who had a formal job before retirement. In rural areas, the initial medical scheme, called the Rural Cooperative Medical Scheme, was implemented from the 1950s to the 1970s during the planned economy. After the collapse of the old system, a new system called the New Cooperative Medical Scheme (NCMS) was launched in the early 2000’s [21, 22]. The URMS and NCMS have now merged into one scheme as of 2016 [16, 17] – allowing rural residents to have the same benefits as urban residents for the first time in Chinese history (although urban older adults who are covered by UEMS benefit from a better healthcare system). In 2011, the enrollment rate for NCMS was approximately 99% -- from less than 10% in 2004. During the same period, enrollment rates for UEMS and URMS reached above 95% in 2010 from less than 20% in the early 2000’s [23–25].

However, studies have shown that one’s access to care is not uniform in China and many older adults report inadequate access to their needed healthcare [5, 7]. Recent research has shown that there were significant differences in access to healthcare between men and women, across age groups, and across urban and rural areas, and that these differences in access to healthcare have significant implications for physical function, cognitive impairment, and overall mortality [5]. However, it is largely unknown how many years of life may be gained or lost due to differences in access to healthcare among older adults in China.

The purpose of this study was to investigate differences in life expectancy associated with access to healthcare at older ages. Using multiple waves of data (2002–2014) from the largest national longitudinal study of adults aged 65 and older in China, we estimated the number of added years of life expectancy associated with reported access to healthcare (adequate vs. inadequate). Analyses adjusted for a wide range of covariates and were presented separately by age, sex, and urban-rural residence. The findings are discussed in the context of a rapidly aging population and their implications for the healthcare system in China and elsewhere.

## Methods

### Data

The study used data from five waves of the Chinese Longitudinal Healthy Longevity Survey (CLHLS) in 2002, 2005, 2008/2009 (hereafter referred to as 2008), 2011/2012 (hereafter as 2011), and 2014. The first two waves of the CLHLS (1998 and 2000) were not used because several key variables were not included and the sample only included adults aged 80 and older at baseline. The CLHLS was conducted in a randomly selected 50% of counties/cities in 22 Han-dominated provinces [26]. The total population of the 22 provinces accounts for approximately 87% of the total population in China in 2010. The CLHLS was designed to oversample very old adults. Thus, sample weights were constructed based on the age-sex-residence-specific distributions of the populations in the sampled provinces and were released with the dataset [27]. The CLHLS has an overall response rate of more than 90% across waves [5, 26].

In 2002, the CLHLS recruited nearly 16,000 older adults. In addition to tracking these older adults, the CLHLS recruited more than 7300 new sample members in 2005, nearly 9000 new sample members in 2008, and nearly 1300 newly old adults in 2011. Consistent with prior research [5, 27], we pooled the five waves of CLHLS data to obtain robust estimates. Specifically, we examined how those who were interviewed in 2005, 2008, and 2011 waves were at risk of dying at 2008, 2011, and 2014 waves. Excluding those who were lost to follow-up, the final analytic sample included 27,794 adults aged 65 and older who were interviewed in 2002–2011.

### Access to healthcare

The National Academies of Sciences, Engineering, and Medicine of the United States defines healthcare (or healthcare) as wide range of services that include preventative care, chronic disease management, emergency services, mental health services, dental care, and other community services that promote health over the lifespan [28]. In the CLHLS, healthcare was defined in the same context and included several questions on the respondents’ access to such healthcare services. In this study, we used the overall question of whether respondents reported having adequate access to healthcare (medical care) services when needed (yes vs. no). On average, about 7.8% of women and 7.4% of men reported having inadequate access to healthcare over the 2002–2014 study period—with some variation based on rural and urban residence (9.0% vs. 4.7%, respectively). To reduce missing data on this question, the CLHLS included responses from proxies (i.e., family members, etc.) to ascertain information on access to healthcare for participants who may be too sick to provide such information. Approximately 23% of sampled older adults used a proxy—with a weighted proportion of only 4.8% using a proxy (because most proxies were for adults aged 90 or older).

### Covariates

The analyses adjusted for a wide range of covariates that have been shown to be associated with either access to healthcare or mortality at older ages [2, 5]. The analyses included a number of demographic, socioeconomic, family/social support, behavioral, and health-related factors that have been shown to be associated with either access to healthcare or mortality [2, 5]. Demographic factors included age (in years), sex, and residence (urban vs. rural). Socioeconomic factors included ethnicity (Han vs. non-Han), years of schooling (0, 1–6, or 7+ years), primary lifetime occupation (white collar vs. other occupations), economic independence (having a retirement wage/pension and/or having own earnings vs. no), and having a health insurance (yes vs. no). Having health insurance referred to whether the respondent was covered by any of the following insurance programs: in addition to abovementioned public free medical scheme, Urban Employer-sponsored Medical Scheme (UEMS), the Urban Resident Medical Scheme (URMS), and the New Cooperative Medical Scheme (NCMS), the CLHLS collected data on whether a respondent purchased severe diseases insurance program. Family/social support included current marital status (married vs. not married) and whether the respondent had close proximity to their children (either co-residing with a child or having a child living in the same village [yes vs. no]).

Health behaviors included whether the respondent was currently smoking (yes vs. no). Health status included (1) physical function, (2) chronic disease, and (3) cognitive impairment. Physical function was measured by disability of activities of daily living (ADLs). ADLs consisted of six activities with three responses: “able to do without help,” “need some help,” and “need full help.” The six items included: (a) bathing, (b) dressing, (c) indoor transferring, (d) toileting, (e) eating, and (f) continence [26]. The six items of ADLs were adopted from the Katz scale and used similar response categories as IADLs [23]. We categorized respondents as ADL disabled (coded as 1) if they reported needing any help in performing any of the six items (otherwise coded as 0). Chronic diseases were ascertained in the CLHLS from 22 major conditions, including hypertension, diabetes, cardiovascular disease, stroke, cancer, and so on. The conditions were self-reported and more than 90% were reported as being diagnosed by a physician. A dichotomous measure for any chronic disease (yes vs. no) was included in the analyses. Cognitive function was measured using the Mini-mental State Examination (MMSE) that includes six domains of cognition—i.e., orientation, reaction, calculation, short-term memory, naming, and language—with a total score of 30. The MMSE items in the CLHLS were adopted from the Folstein MMSE scale [26]. Respondents were categorized as cognitively impaired if his/her MMSE score was below 24 [26]. Given the low level of educational attainment among older adults in China, we assessed alternative criteria (e.g., score of 18) for those with no education to test the sensitivity of different cut-points for defining cognitive impairment. Results were very similar to those presented here and are available upon request. As documented in prior research [26], the Chinese version of MMSE used in the CLHLS was culturally translated from the international standard version of the MMSE questionnaire. The validity and the reliability of the MMSE measure were also carefully tested in pilot surveys and verified in each wave of the CLHLS [26]. With the exceptions of sex, ethnicity, education, and primary lifetime occupation, all measures included in the models were time-varying covariates.

### Mortality and life expectancy

All-cause mortality was defined as whether the respondent was dead or alive at the time of the 2014 survey (event measure). Exposure to mortality risk (duration measure) was ascertained by the number of days alive from the date of the baseline interview to the date of death (for deceased) or the date of the 2014 survey (for survivors). The date of death for deceased respondents was gathered from official death certificates whenever available; otherwise, the information was collected from next-of-kin and confirmed by the local residential committee. From 2002 to 2014, approximately 57.5% of sampled individuals died (it would be 68.3% if those lost to follow-up were excluded) and about 15.8% of respondents were lost to follow-up. The corresponding weighted numbers were 26.7% for death (it would be 31.8% if those lost to follow-up were excluded) and 16.4% for loss to follow. A number of studies have previously documented the high quality of mortality data in the CLHLS [26]. To ensure the reliability of our results, we compared the age-sex-specific death rates from the 2002–2014 CLHLS data to the rates obtained from the 2000 and 2010 Census [26, 29, 30] and the World Population Prospects (WPP) provided by the United Nations Population Division [31]. The high degree of concordance among the death rates from these sources is reported in Figures A1 and A2 in the Additional file 1.

The age-specific death rates were obtained by multiplying the number of time units in the survival analysis relative to a year to the hazard rate estimated from the following formula based on an exponential distribution of the hazard function:

$$ h(x)=\exp \left({\beta}_0+{\beta}_1{x}_1+{\beta}_2{x}_2+{\beta}_3{x}_3+{\beta}_4{x}_4+\sum \limits_{i=1}^K\left({\gamma}_i{z}_i\right)\right), $$

where *β*_0_ is the constant; exp.(*β*_0_) is also referred to as the baseline function. *β*_1_ is the coefficient associated with adequate access to healthcare (*x*_1_); *β*_2_ is the coefficient for age(*x*_2_); and *β*_3_ is the coefficient for sex (*x*_3_). *β*_4_ is the coefficient for urban-rural residence. The four variables *x*_1_, *x*_2_, *x*_3_,and *x*_4_ are all dichotomous variables. For a given sex (*x*_3_), the mean of *x*_4_ is used when the age-specific mortality rates do not distinguish urban-rural differences. In a similar vein, for urban or rural areas (*x*_4_), the mean of *x*_3_ is used when the age-specific mortality rates do not distinguish gender differences. *γ*_*i*_ is the coefficient of covariate *z*_*i*_. *K* is total number of covariates.

In the present study, we estimated the age-sex-specific and the age-residence-specific death rates according to access to healthcare by supplying the coefficients to the different categories of age, sex, urban-rural residence, and access to healthcare, respectively, along with the coefficients of the covariates in the models to their means. We then multiplied 365 to *h*(*x*) to estimate the annual death rate in that the analytical unit of time in our survival analysis is day.

Once the age-sex-specific or age-residence-specific death rates were estimated stratified by adequate and inadequate access to healthcare, life expectancy was calculated for adequate access and for inadequate access stratified by sex or by urban-rural residence using common/basic demographic methods for life tables [32]. The difference in life expectancy between adequate access and inadequate access to healthcare is the increase associated with adequate access or the contribution to life expectancy made by adequate access to healthcare while taking into account the covariates.

### Analytical strategies

Three exponential parametric hazard models were used to examine the difference in life expectancy between adequate access and inadequate access to healthcare or the increase in life expectancy associated with adequate access and how the difference (or advantage) was altered by sex and by urban-rural residence when a wide range of confounding factors were taken into account. Model I included access to healthcare in addition to age, sex, and urban-rural residence. Model II added socioeconomic status to Model I. Model III included all study covariates. Supplementary analyses were also performed to examine whether each of the other three sets of confounding factors (i.e., family/social support, health behaviors, and health conditions) influenced the increase in life expectancy associated with adequate access to healthcare (see Additional file 1: Table A). To ensure the accuracy of model-based death rates in the hazard models, sample weights were always applied. Also, given the different patterns of healthcare utilization, mortality trajectories, and differences by sex and urban-rural residence [33–39], we included interactions between sex and access to healthcare and between urban-rural residence and access to healthcare.

Several sensitivity analyses were also conducted. First, preliminary analyses showed that the results from the parametric hazard models were nearly identical to the estimates from Cox proportional hazard models. Second, preliminary analyses also showed that the results were consistent when assuming that persons lost to follow-up had the same survival status (and length of survival in the survey interval) as those with known survival status if they had the same demographics, psychosocial characteristics, and health conditions. Third, we found no significant interactions between access to healthcare and survey year in our assessment of possible temporal changes in the associations during the 2002–2014 study period. Missing data among all covariates was generally low (less than 2%) and ultimately dropped from the final analyses. Alternative approaches were also assessed (e.g., multiple imputation, mean/mode imputation, etc.) and the results were nearly identical.

## Results

Table 1 shows that the vast majority of older adults in China reported that they could get adequate medical care when needed (weighted, 91–95%). Among the 27,794 CLHLS respondents, the weighted percentage of death was 26.6% in 2002–2014. Overall, women and rural older adults had a lower socioeconomic status than their respective male and urban counterparts. Women were less likely to be married, engage in regular exercise, and smoke than men; whereas women were more likely to be in close proximity to their children and were in poorer health than men. Women also had a lower proportion of death than men. Similar patterns were found in rural areas compared with urban areas—with the exception that rural older adults had better physical functioning. In comparison with older adults who had inadequate access to healthcare, older adults who reported adequate access to healthcare were more likely to be younger, live in urban areas, have a higher socioeconomic status, have higher family/social support, exercise regularly, and have overall better health statuses.

**Table 1:** Distributions of the sample, CLHLS 2002–2014

		Sex		Residence		Access to healthcare	
	Total	Women	Men	Rural	Urban	Inadequate	Adequate
# Sampled individuals	27,794	16,145	11,649	16,929	10,865	2877	24,917
% Adequate access to healthcare	92.4	92.2	92.6	91.0	95.3*	–	–
% Death in 2002–2014 +	26.6	25.3	28.0#	29.3.0	21.7*	33.70	23.2*
Demographics							
Age (mean, years)	71.8	72.0	71.3*	71.9	71.6$	73.8	71.7#
% Men	49.6	–	–	49.9	49.1	48.3	49.7
% Urban	32.0	32.3	31.6	–	–	19.5	33.0*
Socioeconomic factors							
% Han ethnicity	93.2	92.9	93.4	92.2	95.2*	92.8	93.2
% 0 years of schooling	46.4	66.7	26.0*	52.0	34.6*	61.3	45.2*
% 6 years of schooling	38.3	26.4	50.4*	38.3	38.4*	32.4	38.8*
% 7+ years of schooling	15.3	7.0	23.7*	9.7	27.0*	6.3	16.0*
% Economic independence	52.2	42.0	62.6*	44.8	67.8*	46.8	52.6#
% Professional occupation	13.0	10.9	15.2*	7.3	25.3*	6.7	13.6*
% Having a health insurance	34.2	31.4	37.1*	34.0	34.7	22.3	35.2*
Family/Social support							
% Married	65.3	53.3	77.5*	64.4	67.1$	48.2	66.7*
% Close proximity to children	84.3	85.9	82.7#	88.0	76.5*	75.2	85.1*
Health practice							
% Doing regular exercise	32.9	29.1	36.8*	23.5	53.0*	20.8	33.9*
% Currently smoking	27.2	7.9	46.8*	27.6	26.4	29.6	27.0
Health condition							
% ADL disabled	6.1	7.2	5.0*	5.6	7.1#	9.4	5.8*
% Cognitively impaired	11.9	16.2	7.6*	13.3	9.0*	26.0	10.8*
% Having 1+ chronic disease	57.9	60.1	55.6*	54.6	64.9*	63.6	57.4#
Waves							
% Samples in 2002	48.0	21.5	46.0#	48.0	47.8*	53.2	47.5#
% Samples in 2005	18.0	17.8	18.3#	15.8	22.7*	19.4	17.9#
% Samples in 2008	24.1	23.0	25.2#	23.3	25.7*	20.5	24.4#
% Samples in 2011	9.9	9.3	10.5#	12.8	3.8*	6.9	10.2#

Note: (1) The percentages were weighted and calculated from the respondents excluding those lost to follow-up. The weighted percentage distribution reflected the characteristics of the respondents at their first interviews. The distribution is very similar if those lost to follow-up were included. (2) +, the weighted percentages of death were calculated from number of deaths among initially total interviewed sample, including those lost to follow-up. (3) The tests for percentage distribution between women and men, between rural and urban, and between inadequate and adequate access to healthcare were based on Wald test. $, *p* < 0.05, #, *p* < 0.001, *, *p* < 0.001

**Fig. 1:**
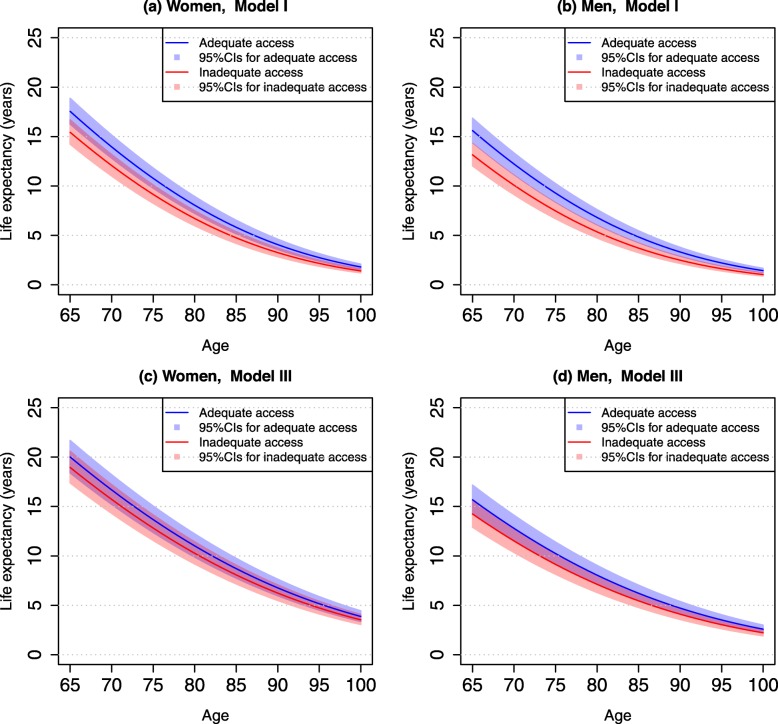
Estimated Life Expectancy (95% Confidence Intervals) Associated with Adequate and Inadequate Access to Healthcare by Age and Sex, CLHLS 2002–2014

**Fig. 2:**
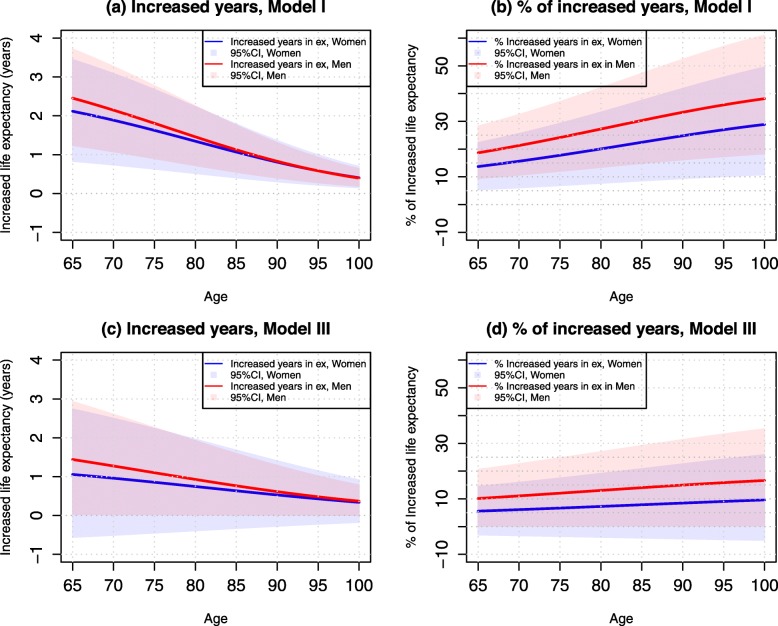
Increased Years in Life Expectancy (95% Confidence Intervals) and Their Percentages Associated with Adequate Access vs. Inadequate Access to Healthcare by Age and Sex CLHLS 2002–2014

Table 2 presents life expectancies associated with adequate and inadequate access to healthcare by age for men and women. Overall, life expectancy at age 65 was 17.42 years for women and 15.46 years for men. At age 85, life expectancy was 5.73 years for women and 4.75 years for men. After taking into account demographic background (Model I), adequate access to healthcare increased life expectancy at age 65 by approximately 2.12 years (95% CI: 0.83–3.44) in women and 2.46 years (95% CI: 1.24–3.72) in men. At age 85, the corresponding increases in life expectancy were 1.07 years (95% CI: 0.41–1.78) in women and 1.12 years (95% CI: 0.65–1.75) in men. However, the relative increases (advantage)—i.e., the percentage difference in life expectancy between adequate access and inadequate access—were higher at age 85 than at age 65. Thus, we found that adequate access to healthcare had a greater advantage for men than for women in terms of more years of life. Figure 1 further illustrates the differences in life expectancy from ages 65–100 by sex in those who reported adequate access to healthcare compared with inadequate access to healthcare. Figure 2 illustrates the increases in life expectancy (in years) and their percentage increases attributable to adequate access to healthcare.

**Table 2:** Life expectancy at ages 65 and 85 by access to healthcare and sex, CLHLS 2002–2014

	Women	Men
Life expectancy at age 65 (years) ^a^	17.42	15.46
Life expectancy at age 85 (years) ^a^	5.73	4.75
**Model I**		
Age 65		
Inadequate access to care (I) (years)	15.43 (14.22–16.68)	13.15(12.04–14.32)
Adequate access to care (A) (years)	17.55 (16.26–18.87)	15.61 (14.39–16.87)
Difference in LE (A-I) (years)	2.12 (0.83–3.44)	2.46 (1.24–3.72)
% of difference (A-I)/I	13.74 (5.38–22.29)	18.71 (9.43–28.29)
Age 85		
Inadequate access to care (I) (years)	4.75 (4.18–5.37)	3.71 (3.24–4.23)
Adequate access to care (A) (years)	5.82 (5.16–6.53)	4.83 (4.36–5.46)
Difference in LE (A-I) (years)	1.07 (0.41–1.78)	1.12 (0.65–1.75)
% of difference (A-I)/I	22.53 (8.63–37.47)	30.19 (17.52–47.17)
**Model II**		
Age 65		
Inadequate access to care (I) (years)	16.19 (14.92–17.52)	13.06 (11.92–14.25)
Adequate access to care (A) (years)	18.28 (16.93–19.67)	15.39 (14.35–16.68)
Difference in LE (A-I) (years)	2.09 (0.74–3.48)	2.33 (1.29–3.62)
% of difference (A-I)/I	12.91 (4.57–31.49)	17.84 (9.88–27,72)
Age 85		
Inadequate access to care (I) (years)	5.39 (4.76–6.08)	3.90 (3.41–4.44)
Adequate access to care (A) (years)	6.49 (5.77–7.28)	4.99 (4.40–5.64)
Difference in LE (A-I) (years)	1.10 (0.38–1.89)	1.09 (0.50–1.74)
% of difference (A-I)/I	20.41 (7.05–35.06)	27.95 (12.82–44.62)
**Model III**		
Age 65		
Inadequate access to care (I) (years)	18.95 (17.38–20.59)	14.24 (12.91–15.66)
Adequate access to care (A) (years)	20.01 (18.39–21.69)	15.68 (14.27–17.17)
Difference in LE (A-I) (years)	1.06 (−0.56–2.74)	1.44 (0.03–2.93)
% of difference (A-I)/I	5.59 (−2.96–14.46)	10.11 (0.21–20.58)
Age 85		
Inadequate access to care (I) (years)	8.07 (7.16–9.07)	5.45 (4.77–6.20)
Adequate access to care (A) (years)	8.71 (7.74–9.76)	6.22 (5.47–7.04)
Difference in LE (A-I) (years)	0.64 (−0.33–1.69)	0.77 (0.02–1.59)
% of difference (A-I)/I	7.93 (−4.09–20.94)	14.13 (0.37–29.17)

^a^ Life expectancy was estimated from models that included age, sex, and urban-rural residence only

Model I included whether a respondent could get adequate access to medical care, adjusting for age, sex, and urban-rural residence. Model II added socioeconomic variables and year of survey to Model I. Model III included all study variables in Table 1. Numbers in the parentheses are 95% CIs

After adjustment for multiple socioeconomic factors (Model II), life expectancies were mildly “improved” among women and among the oldest-old—suggesting that individuals who report inadequate access to care can be partially attributed to their respectively lower socioeconomic statuses. As a result, the difference in life expectancy between adequate and inadequate access to healthcare was attenuated. After adjustment for all study covariates (Model III), life expectancies among women, the oldest-old, and among persons with inadequate access to healthcare were further “improved;” and the increases (advantages) in life expectancies associated with adequate access to care were further reduced. Compared to those who reported inadequate access to healthcare, we found that adequate access to healthcare was associated with 1.06 years (95% CI: − 0.56-2.74) of longer life expectancy at age 65 in women and 1.44 years (95% CI: 0.03–2.93) of longer life expectancy in men. At age 85, the corresponding life expectancies improved by 0.64 years (95% CI: − 0.33-1.69) in women and 0.77 years (95% CI: 0.02–1.59) in men. In terms of relative increases, we found that life expectancies increased by approximately 6–10% at age 65 and 8–14% at age 85. Overall, the number of years and relative increases in life expectancies due to adequate access to healthcare were generally larger among men than among women—and these differences were largest at the oldest-old ages.

Table 3 presents life expectancies associated with access to healthcare at ages 65 and 85 by urban-rural residence. Results show that differences in life expectancy between urban and rural older adults was small (0.30 years at age 65 and 0.18 years at age 85). However, we found that life expectancies associated with adequate vs. inadequate access to healthcare at age 65 were 2.04 years (95% CI: 0.81–3.33) in rural areas and 2.14 years (95% CI: 0.87–3.45) in urban areas. At age 85, the corresponding improvement in life expectancies was 0.99 years (95% CI: 0.38–1.64) in urban areas and 1.06 years (95% CI: 0.42–1.75) in rural areas. The relative percentage of increase in life expectancy was nearly the same in urban and rural areas.

**Table 3:** Life expectancy at ages 65 and 85 by access to healthcare and urban-rural residence, CLHLS 2002–2014

	Rural	Urban
Life expectancy at age 65 (years)^a^	16.10	16.48
Life expectancy at age 85 (years)^a^	5.06	5.24
**Model I**		
Age 65		
Inadequate access to care (I) (years)	14.25 (13.09–15.46)	14.97 (13.78–16.21)
Adequate access to care (A) (years)	16.29 (15.06–17.58)	17.11 (15.84–18.42)
Difference in LE (A-I) (years)	2.04 (0.81–3.33)	2.14 (0.87–3.45)
% of difference (A-I)/I	14.32 (5.68–23.37)	14.30 (5.81–23.05)
Age 85		
Inadequate access to care (I) (years)	4.19 (3.68–4.77)	4.53 (3.99–5.13)
Adequate access to care (A) (years)	5.18 (4.57–5.83)	5.59 (4.95–6.28)
Difference in LE (A-I) (years)	0.99 (0.38–1.64)	1.06 (0.42–1.75)
% of difference (A-I)/I	23.63 (9.07–39.14)	23.40 (9.27–38.63)
**Model II**		
Age 65		
Inadequate access to care (I) (years)	14.80 (13.58–16.07)	15.23 (13.99–16.52)
Adequate access to care (A) (years)	16.80 (15.51–18.15)	16.77 (15.98–18.11)
Difference in LE (A-I) (years)	2.00 (0.71–3.35)	1.54 (0.75–2.88)
% of difference (A-I)/I	13.51 (4.80–22.64)	10.11 (4.92–18.91)
Age 85		
Inadequate access to care (I) (years)	4.70 (4.13–5.33)	4.91 (4.32–5.56)
Adequate access to care (A) (years)	5.70 (5.05–6.42)	5.69 (5.03–6.40)
Difference in LE (A-I) (years)	1.00 (0.35–1.72)	0.78 (0.12–1.49)
% of difference (A-I)/I	21.28 (7.45–36.60)	15.89 (2.44–30.35)
**Model III**		
Age 65		
Inadequate access to care (I) (years)	16.80 (15.33–18.35)	17.31 (15.81–18.88)
Adequate access to care (A) (years)	17.80 (16.28–19.39)	17.70 (16.19–19.28)
Difference in LE (A-I) (years)	1.00 (−0.52–2.59)	0.39 (−1.12–1.97)
% of difference (A-I)/I	5.95 (−3.10–15.42)	2.25 (−6.47–11.38)
Age 85		
Inadequate access to care (I) (years)	6.84 (6.03–7.72)	7.12 (6.29–8.03)
Adequate access to care (A) (years)	7.40 (6.54–8.33)	7.34 (6.49–8.27)
Difference in LE (A-I) (years)	0.56 (−0.3–1.49)	0.22 (−0.63–1.15)
% of difference (A-I)/I	8.19 (−0.39–21.78)	3.09 (−8.85–16.15)

^a^Life expectancy was estimated from models that included age, sex, and urban-rural residence only

Model I included whether a respondent could get adequate access to medical care, adjusting for age, sex, and urban-rural residence. Model II added socioeconomic variables and year of survey to Model I. Model III included all study variables in Table 1. Numbers in the parentheses are 95% CIs

After adjustment for multiple socioeconomic factors (Model II), life expectancy “decreased” in urban areas and “increased” in rural areas—largely due to the socioeconomic disadvantages in rural areas. Thus, we found that life expectancy among rural adults who had adequate access to healthcare was slightly higher than the life expectancy among their urban counterparts. With regard to the increase in life expectancy associated with adequate access to healthcare, we found that it was only slightly reduced in rural areas after taking into account socioeconomic factors (from Model I to Model II); whereas it was substantially reduced in urban areas.

**Fig. 3:**
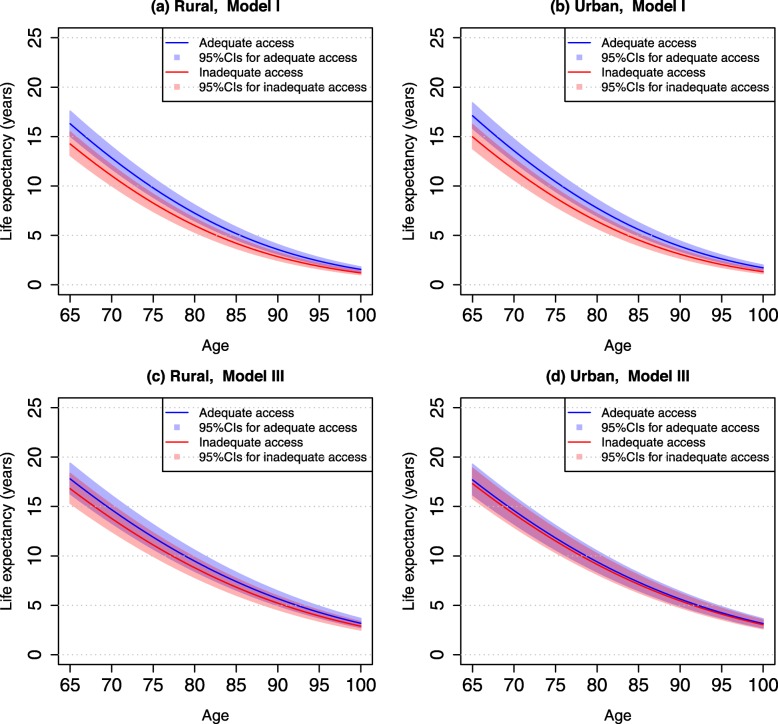
Estimated Life Expectancy (95% Confidence Intervals) Associated with Adequate and Inadequate Access to Healthcare by Age and Urban-rural Residence, CLHLS 2002–2014

**Fig. 4:**
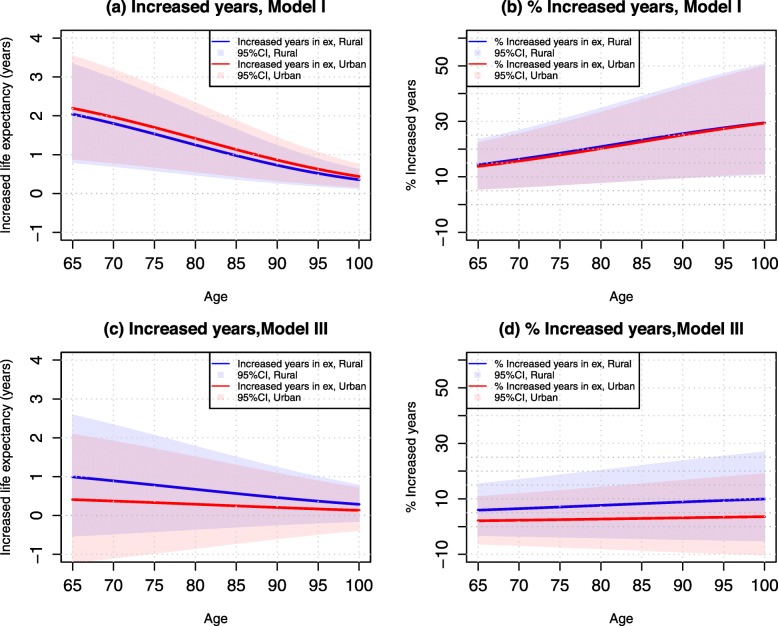
Increased Years in Life Expectancy (95% Confidence Intervals) and Their Percentages Associated with Adequate Access vs. Inadequate Access to Healthcare by Age and Urban-Rural Residence, CLHLS 2002–2014

After adjustment for all study covariates (Model III), life expectancy among rural older adults was further “improved” – suggesting that they are generally disadvantaged in terms of their family/social support, health practices, and health status relative to urban older adults. Accordingly, the increases in life expectancy associated with adequate access to healthcare were further reduced to 1.0 years at age 65 and 0.56 years at age 85 in rural areas (or 6 and 8% in relative terms). The increase in years of life expectancy due to adequate access to healthcare was comparatively small in urban areas—0.39 years at age 65 (~ 2%) and 0.22 years age 85 (~ 3%). Figure 3 further illustrates the differences in life expectancy from ages 65–100 by urban-rural residence in those who reported adequate access to healthcare compared with inadequate access to healthcare. Figure 4 illustrates the increases in life expectancy (in years) and their percentage increases attributable to adequate access to healthcare.

Finally, our supplementary analyses showed that the overall life expectancy patterns associated with access to healthcare by sex and urban-rural residence were similar when different sets of covariates were adjusted for separately. In general, health conditions had a greater impact on differences in life expectancy between in/adequate access to healthcare than health practices and family/social support; and that health practices (smoking) played a stronger role than family/social support (see Table A in Additional file 1).

## Discussion

Studies have shown that access to healthcare is strongly associated with the health and mortality of older adults [3, 5, 7]. Using the largest national longitudinal study of older adults in China, we found that adequate access to health increased life expectancy by 2.0–2.5 years at age 65 and 1.0–1.2 years at age 85 compared with having inadequate access to healthcare. However, the increases in life expectancy were largely attenuated after accounting for a wide range of factors, including socioeconomic status, family/social support, health practices, and health status. The attenuation was largely attributable to the lower socioeconomic status, poorer family/social support, and poorer health status among those who had inadequate access to healthcare. Net of these confounding factors, we found that adequate access to healthcare increased life expectancy at older ages by 6–8% in women and by 10–14% in men. We also found that adequate access to healthcare increased life expectancy at older ages by 2–3% in urban areas and by 6–8% in rural areas.

Overall, the improvements in life expectancy associated with adequate access to healthcare were slightly larger among men than among women. This finding was not entirely unexpected. Although women are more likely to use preventive care, outpatient care, and community/home-based services than men [38], studies show that women often receive fewer inpatient services and less aggressive treatments than men [37]. Furthermore, older women generally have less educational attainment and fewer economic resources than older men [39]. This is especially the case for older women in China who may have experienced sexism and other cultural constraints at younger ages in this traditional social system [40]. Together, the greater need for care and the absence of resources may lead to delays in healthcare—and/or lower-quality care—that may exacerbate health decline in women [8, 9].

One notable finding was that the increases in life expectancy (in years) associated with adequate access to healthcare were slightly lower in rural areas than in urban areas. However, when socioeconomic factors and/or other confounding factors were taken into account, the increase in life expectancy associated with adequate access to healthcare was greater in rural areas than in urban areas. This finding is consistent with recent research showing somewhat greater reductions in mortality related to adequate access to healthcare in rural settings than in urban settings [5]. Although the healthcare system was improved in rural China in 2000–2015 [41], these areas continued to have healthcare programs that were less adequate than those in urban areas [25, 35, 42]. It has been shown that financial barriers are often major obstacles for acquiring care in rural areas [5]; largely because of low income (and few pensions), high out-of-pocket costs, and high medical co-payments [43]. As a result, rural older adults face disproportionately greater difficulties in accessing their medical services when needed [5].

Another notable finding was that although the absolute gains in life expectancy (i.e., years) related to access to healthcare decreased with age, the relative gains in life expectancy (i.e., % years lived) increased with age. With lower overall life expectancy at the oldest-old ages, it is reasonable to expect than even small improvements in the number of years lived can translate into comparatively large gains in relative life expectancy. Likewise, oldest-old adults are more physically frail and thus may be more vulnerable to their external environment [44]. Moreover, much like the differences by urban-rural residence, we found that adjustment for multiple covariates attenuated these differences across age. This suggests that the observed differences in life expectancy were also largely attributable to the socioeconomic, family/social support, behavioral, and health-related characteristics of older adults in China. The associations among these factors and mortality have been well-documented [5, 27, 45], and likewise, have been recently shown to explain some of the association between access to healthcare and mortality in this population [5]. However, more research is needed to fully assess the role of these factors and further identify other factors that may play a role.

When examining how adequate access to healthcare contributes to life expectancy, we also found that the increase in life expectancy among women relative to men was enhanced after taking into account socioeconomic factors, health status, and other confounding factors. We suspect this is because women generally have lower socioeconomic status and poorer health conditions than men [27, 35, 37]. Thus, when controlling for these factors, women’s longevity is expected to increase. Similarly, the lower life expectancy of rural older adults was attenuated, and even reversed, when major confounding factors (namely socioeconomic status) were included in the models [19, 33]. Nevertheless, the increases in life expectancy associated with healthcare persisted by sex and urban-rural residence despite multiple covariate adjustments.

A key strength of our study was the use of a large nationally representative longitudinal sample of older adults in China from 2002 to 2014. Using multiple waves of follow-up data, we were able to model group differences in life expectancy while accounting for concurrent (time-varying) changes in a wide range of individual-level factors. In doing so, we further extend the contributions of recent studies that have demonstrated associations between access to healthcare and various health outcomes in China [5]. In addition, we examine these time-varying associations during an important transitional period in China that witnessed dramatic changes in the coverage and accessibility of healthcare—particularly in rural areas following the collapse of an older cooperative medical scheme in the early 2000s [5, 16–21].

Another key contribution of our study was the use of self-reported access to healthcare. There is a growing body of research that has shifted the use of objective measures of utilization of healthcare at older ages to the use of self-reported measure to mitigate the counterintuitive findings [7, 46]. For example, some have argued that the actual utilization is an endogenous factor may confound the observed association between healthcare and health outcomes [47]—i.e., people with complex comorbidities exhibit higher rates of utilization. Likewise, there has been a debate about the bi-direction association between health insurance coverage and health status—i.e., whether having insurance impacts health or whether health status impacts having insurance [48]. The current study minimized these issues of endogeneity and captured important information beyond the utilization [48, 49]. Self-reported access to medical care reflects an individual’s wider context and perceptions about whether they can obtain healthcare services when needed—including information about (i) whether the use of healthcare meets their needs, (ii) whether they could get timely treatment, (iii) whether there are any barriers or delays in receiving care, (iv) whether the services they received are satisfactory, and (v) other perceived dimensions in accessing care [5, 48, 49].

Several limitations of the study should be noted. First, self-reported access to healthcare may not reflect actual use of healthcare and may be associated with individual factors such as demographic background, socioeconomic status, health literacy, health status, and prior utilization [2, 48]. Although we adjusted for many of these factors, additional approaches (e.g., “anchoring vignettes”) have been proposed to address this issue [50]. With no other surveys in China that have collected data on self-reported access to healthcare, more research is clearly warranted to further elucidate its conceptual and substantive implications. Second and relatedly, the CLHLS lacked data on barriers to healthcare (e.g., lack of transportation, distance, or travel terrain), the availability of specific healthcare services, episodic experience and timeframe of doctor visits and hospitalization, and the quality of healthcare services—which may influence reports of one’s access and/or use of healthcare [9]. Therefore, we were unable to determine whether the associations reported in this study were independent of actual availability or use of healthcare [5]. Third, although the CLHLS had somewhat higher-quality mortality data than the censuses (as shown in the Additional file 1), it is not immune from undercounts of death—particularly in rural areas—and we recognize that it may introduce bias in our analyses. Fourth, previous studies have demonstrated the influence of contextual factors on access to healthcare and mortality—such as the level of neighborhood socioeconomic status and development, availability of healthcare facilities in a community, number of medical professionals within the facilities, etc. [51, 52]. However, due to the lack of data on such measures in the CLHLS, we were unable to directly model the effects of these potential contextual factors. Therefore, we encourage future research to consider these important factors. Finally, although we took into account a wide range of individual-level covariates, we recognize that other contextual factors may be related to differences in mortality. Therefore, more research is needed to investigate how contextual characteristics (e.g., geography, local economy, community resources, etc.) may be contributing to the association between reported access to healthcare and survival.

Despite these limitations, our study provided new evidence to demonstrate the sizeable and robust increases in life expectancy associated with having adequate access to healthcare. Moreover, we showed that these improvements were related to sex and urban-rural residence, and were differentially influenced by a wide array of confounding factors. Collectively, these findings underscore the importance of improving access to healthcare to promote longevity and reduce the burden of disease [7]. Furthermore, based on our findings of how specific covariates contributed to the association between access to healthcare and life expectancy, it is important to target more directly older adults who report inadequate access to healthcare and likewise face disadvantages in their socioeconomic status, social/family support, and health status to help close the gap from those with adequate access to their healthcare. These findings also have important policy implications for achieving sustainable development goals that can actively promote healthy lives and well-being for older adults as stated in the UN’s 2030 agenda [53].

## Conclusions

In sum, the current findings have potentially important implications for efforts to improve access to healthcare in older populations, especially in the era of Sustainable Development Goals [7]. In the context of nearly-universal healthcare coverage in China, we found that some older adults still reported inadequate access to their needed healthcare. Although state-sponsored health insurance initiatives have made substantial improvements in providing coverage [9]; there remain challenges in providing adequate access to medical services throughout urban and rural settings because of rapid population aging, the skyrocketing costs of medications and medical services, an increasing shortage of healthcare professionals, and reduced resources to support familial caregiving [5]. Recent research has suggested that over half of older adults in China who did not seek outpatient services attribute it to the affordability of medical costs [5]; and many others attribute it to transportation issues or an overly-complicated reimbursement process. Therefore, addressing these barriers to care will be critical for achieving Sustainable Development Goals that further improve access to healthcare and the commensurate advantages in life expectancy that it has been shown to afford.

## Supplementary information

**Additional file 1: Figure A1**. Comparison of death rates between CLHLS, Censuses, the UN World Population Prospects, Women. **Figure A2**. Comparison of death rates between CLHLS, Censuses, and the UN World Population Prospects, Men. **Table A**. Life Expectancy (95% Confidence Intervals) at Ages 65 and 85 by Access to Healthcare for Women, Men, Rural, and Urban Older Adults, CLHLS 2002-2014.

## Data Availability

The CLHLS datasets are publicly available at the National Archive of Computerized Data on Aging, University of Michigan (http://www.icpsr.umich.edu/icpsrweb/NACDA/studies/36179). Researchers can obtain these data after submitting a data use agreement to the CLHLS team.

## Abbreviations

ADL: Activities of daily living
CLHLS: Chinese Longitudinal Healthy Longevity Survey
MMSE: Mini-mental state examination
NCMS: New Cooperative Medical Scheme
UEMS: Urban Employer-sponsored Medical Scheme
URMS: Urban Resident Medical Scheme
