# Supplementary material for: Adequate access to healthcare and added life expectancy among older adults in China
Source: BMC Geriatr. 2020 Apr 9;20:129. doi: 10.1186/s12877-020-01524-9 (PMC7146971; doi:10.1186/s12877-020-01524-9)
Supplement: Supplementary file 1 — Additional file 1: Figure A1. Comparison of death rates between CLHLS, Censuses, the UN World Population Prospects, Women. Figure A2. Comparison of death rates between CLHLS, Censuses, and the UN World Population Prospects, Men. Table A. Life Expectancy (95% Confidence Intervals) at Ages 65 and 85 by Access to Healthcare for Women, Men, Rural, and Urban Older Adults, CLHLS 2002-2014. [file 12877_2020_1524_MOESM1_ESM.doc]

**Appendix**

**Figure A1. Comparison of death rates between CLHLS, Censuses, the UN World Population Prospects, Women**

Note: The 2002-2014 CLHLS refers to the entire period (which may refer to year 2008). The 2000-2010 censuses were weighted to reflect the year 2008. The data for UN World Population Prospects refer to the year 2008 (which was obtained from the 2019 revision).

**Figure A2. Comparison of death rates between CLHLS, Censuses, and the UN World Population Prospects, Men**

Note: The 2002-2014 CLHLS refers to the entire period (which may refer to year 2008). The 2000-2010 censuses were weighted to reflect the year 2008. The data for UN World Population Prospects refer to the year 2008 (which was obtained from the 2019 revision).

**Table A. Life Expectancy (95% Confidence Intervals) at Ages 65 and 85 by Access to Healthcare for Women, Men, Rural, and Urban Older Adults, CLHLS 2002-2014**

|  | Age 65 | | |  | Age 85 | | |
| --- | --- | --- | --- | --- | --- | --- | --- |
|  | Model A | Model B | Model C |  | Model A | Model B | Model C |
|  |  |  |  |  |  |  |  |
| **Women** |  |  |  |  |  |  |  |
| Inadequate access to care (I) (years) | 16.00 (14.76-17.29) | 15.99 (14.76-17.25) | 17.97 (16.52-19.48) |  | 5.15 (4.55-5.81) | 5.02 (4.43-5.66) | 7.01 (6.21-7.88) |
| Adequate access to care (A) (years) | 17.91 (16.60-19.26) | 17.75 (16.47-19.07) | 18.87 (17.38-20.41) |  | 6.13 (5.45-6.88) | 5.92 (5.26-6.64) | 7.52 (6.68-8.44) |
| Difference in LE (A-I) (years) | 1.91 (0.60-3.26) | 1.76 (0.48-3.08) | 0.90 (-0.59-2.44) |  | 0.98 (0.30-1.73) | 0.90 (0.24-1.62) | 0.51 (-0.33-1.43) |
| % of difference (A-I)/I | 11.94 (3.75-20.38) | 11.01 (3.00-19.26) | 5.01 (-3.28-13.58) |  | 19.03 (5.83-33.59) | 17.93 (4.78-32.27) | 7.28 (-4.71-20.4) |
| **Men** |  |  |  |  |  |  |  |
| Inadequate access to care (I) (years) | 13.23 (12.10-14.41) | 13.28 (12.17-14.44) | 14.32 (13.03-15.66) |  | 3.85 (3.37-4.38) | 3.76 (3.29-4.28) | 5.06 (4.44-5.76) |
| Adequate access to care (A) (years) | 15.46 (14.24-16.73) | 15.35 (14.15-16.59) | 15.87 (14.51-17.29) |  | 4.88 (4.30-5.52) | 4.71 (4.15-5.31) | 5.86 (5.17-6.63) |
| Difference in LE (A-I) (years) | 2.23 (1.01-3.50) | 2.07 (0.87-3.31) | 1.55 (0.19-2.97) |  | 1.03 (0.45-1.67) | 0.95 (0.39-1.55) | 0.80 (0.11-1.57) |
| % of difference (A-I)/I | 16.86 (7.63-26.46) | 15.59 (6.55-24.92) | 10.82 (1.33-20.74) |  | 26.75 (11.69-43.38) | 25.27 (10.37-41.22) | 15.81 (2.17-31.03) |
| **Rural** |  |  |  |  |  |  |  |
| Inadequate access to care (I) (years) | 14.65 (13.46-15.89) | 14.78 (13.61-16.00) | 16.19 (14.81-17.62) |  | 4.50 (3.95-5.09) | 4.44 (3.90-5.02) | 6.03 (5.32-6.82) |
| Adequate access to care (A) (years) | 16.48 (15.21-17.78) | 16.48 (15.24-17.76) | 17.04 (15.63-18.51) |  | 5.39 (4.77-6.07) | 5.26 (4.66-5.93) | 6.49 (5.74-7.32) |
| Difference in LE (A-I) (years) | 1.83 (0.56-3.13) | 1.70 (0.46-2.98) | 0.85 (-0.56-2.32) |  | 0.89 (0.27-1.57) | 0.82 (0.22-1.49) | 0.46 (-0.29-1.29) |
| % of difference (A-I)/I | 12.49 (3.82-21.37) | 11.5 (3.11-20.16) | 5.25 (-3.46-14.33) |  | 19.78 (6.00-34.89) | 18.47 (4.95-33.56) | 7.63 (-4.81-21.39) |
| **Urban** |  |  |  |  |  |  |  |
| Inadequate access to care (I) (years) | 15.11 (13.90-16.36) | 15.06 (13.87-16.29) | 16.95 (15.54-18.42) |  | 4.71 (4.15-5.33) | 4.57 (4.02-5.17) | 6.44 (5.69-7.26) |
| Adequate access to care (A) (years) | 17.03 (15.75-18.35) | 16.60 (15.36-17.88) | 17.93 (16.49-19.44) |  | 5.67 (5.03-6.38) | 5.32 (4.71-5.99) | 6.99 (6.19-7.86) |
| Difference in LE (A-I) (years) | 1.92 (0.64-3.24) | 1.54 (0.30-2.82) | 0.98 (-0.46-2.49) |  | 0.96 (0.32-1.67) | 0.75 (0.14-1.42) | 0.55 (-0.25-1.42) |
| % of difference (A-I)/I | 12.71 (4.24-21.44) | 10.23 (1.99-18.73) | 5.78 (-2.71-14.69) |  | 20.38 (6.79-35.46) | 16.41 (3.06-31.07) | 8.54 (-3.88-22.05) |
| Note: Model A adjusted for age, sex, urban-rural residence, family/social support, and year of survey. Model B adjusted for age, sex, urban-rural residence, health practices, and year of survey. Model C adjusted for age, sex, urban-rural residence, health conditions, and year of survey. | | | | | | | |
